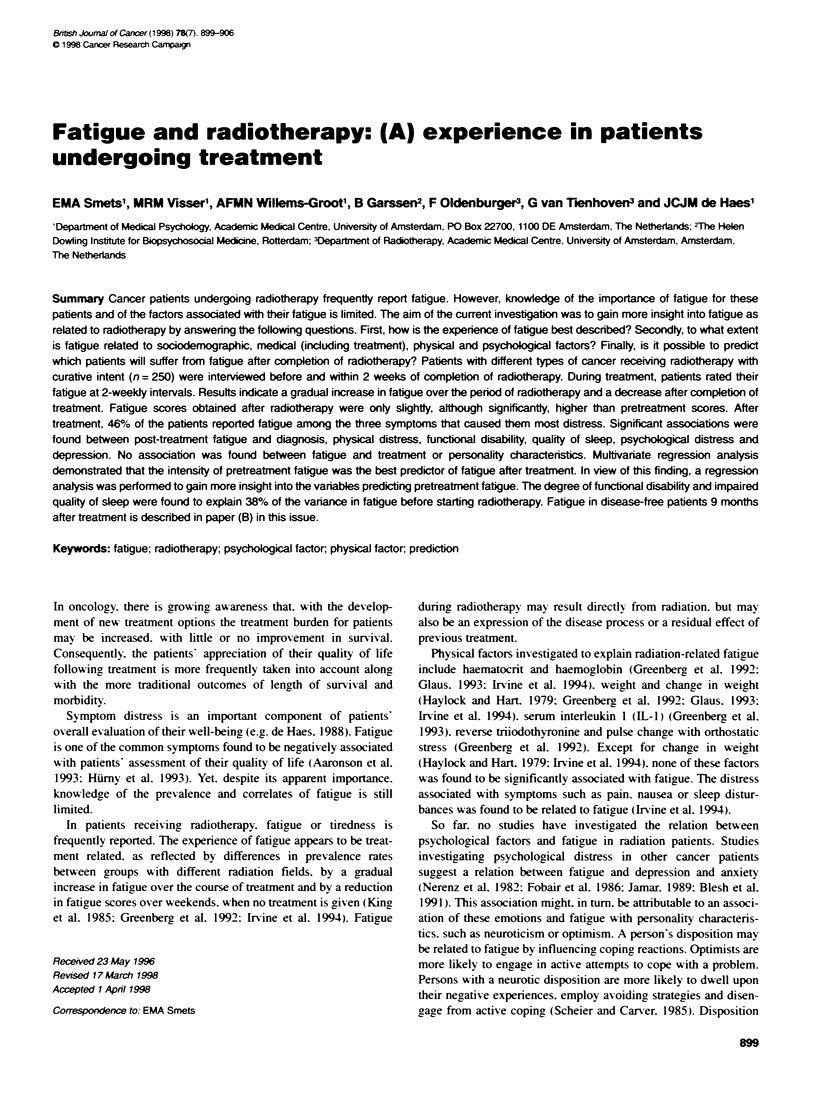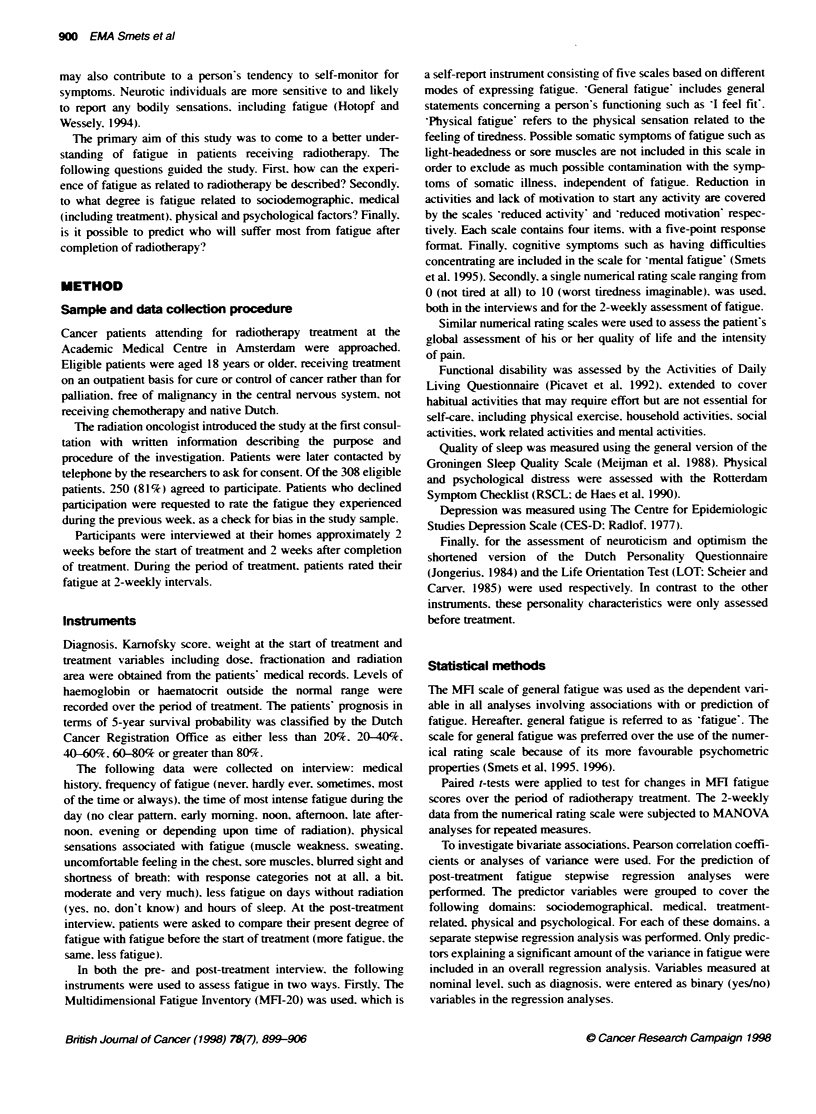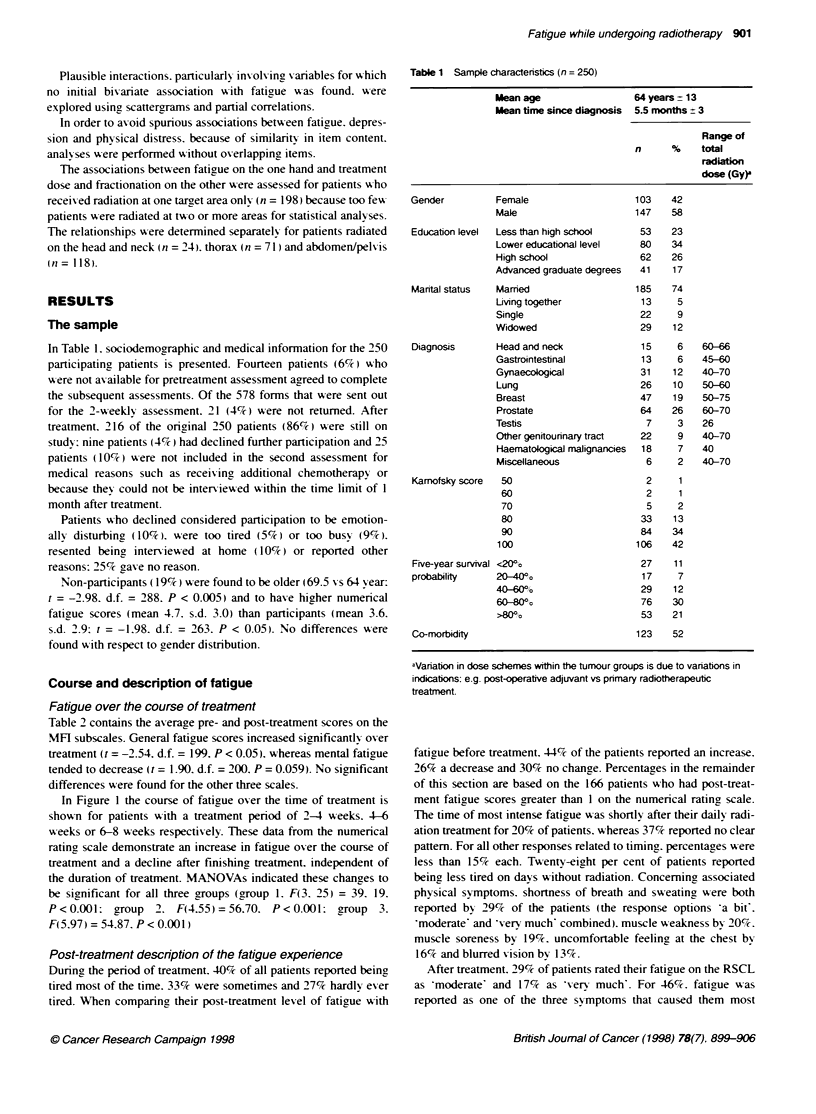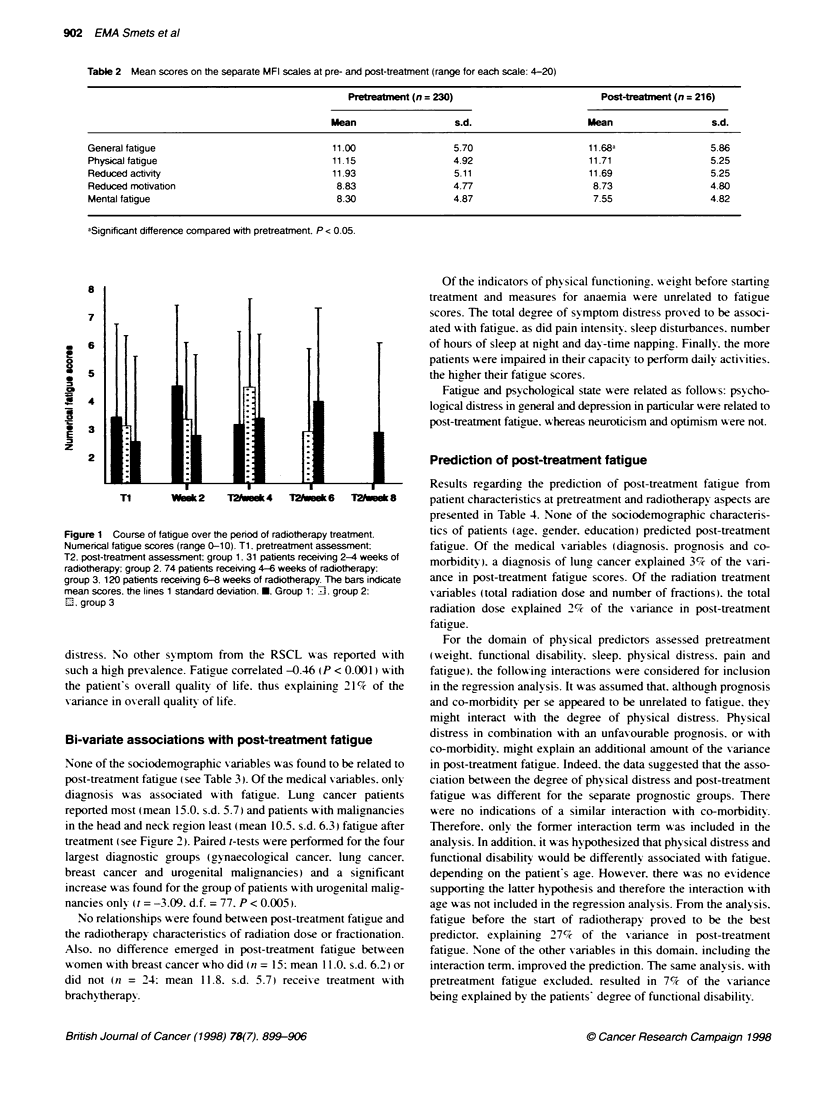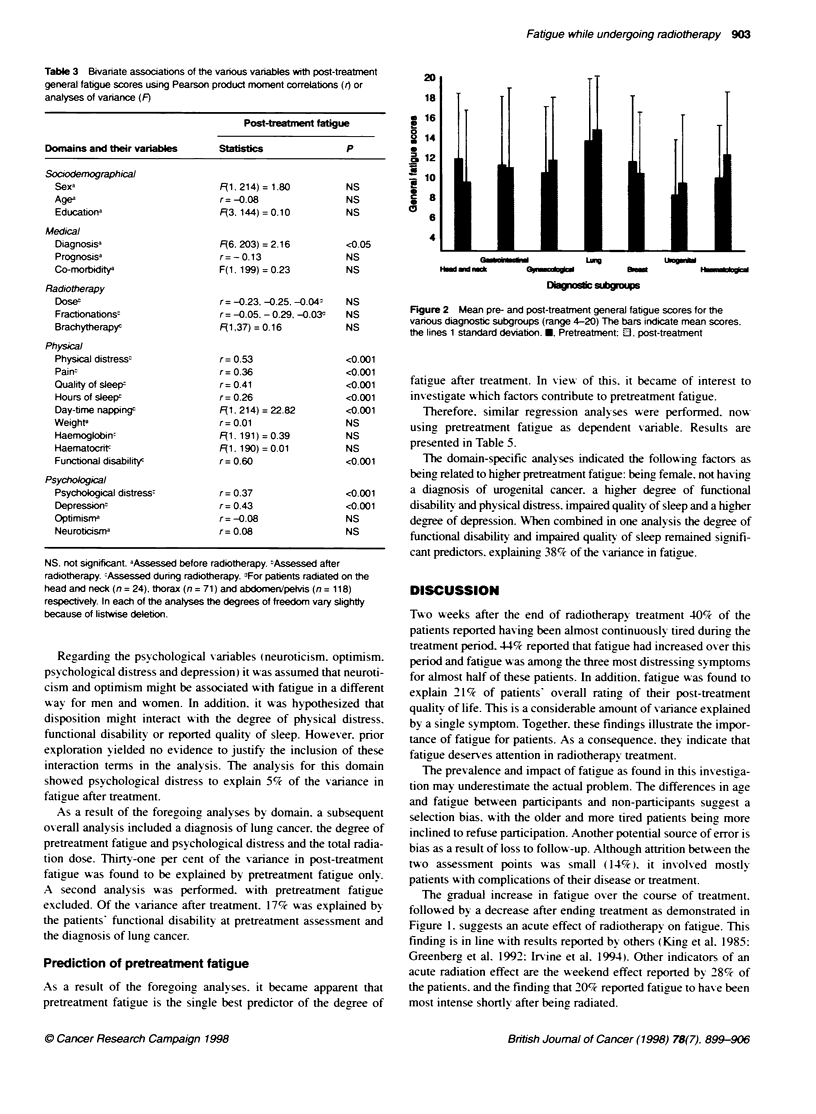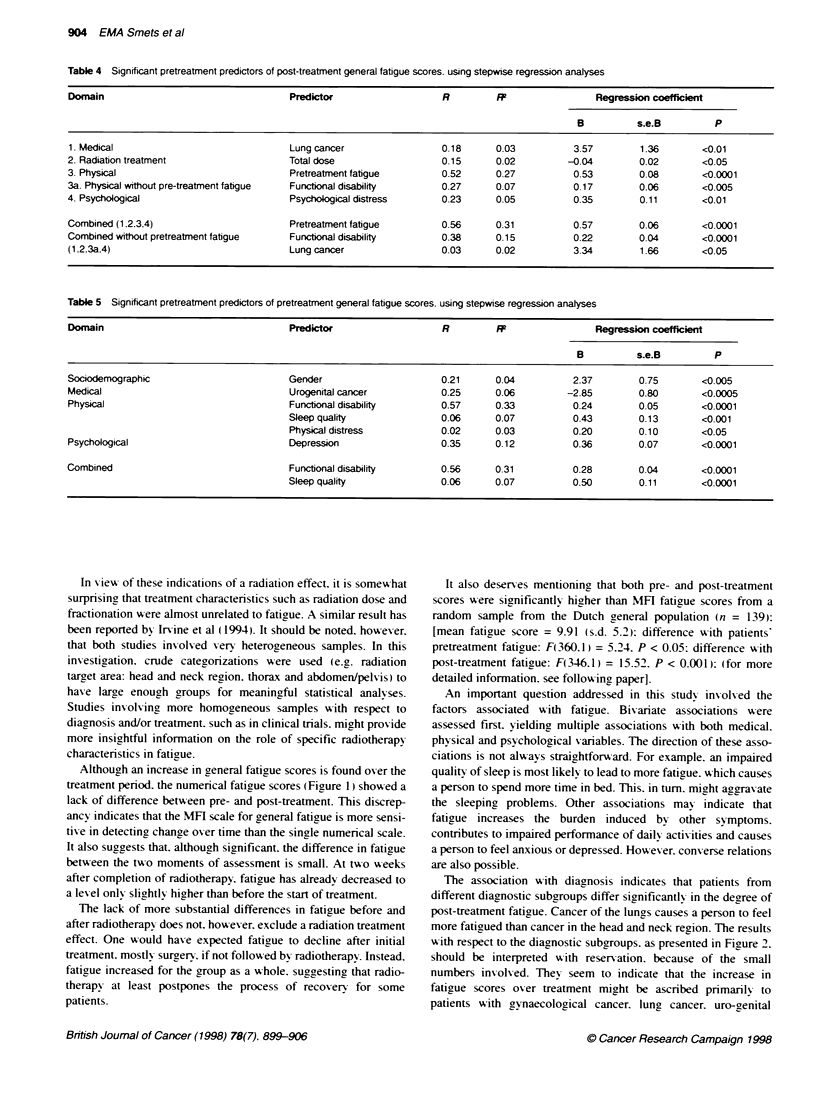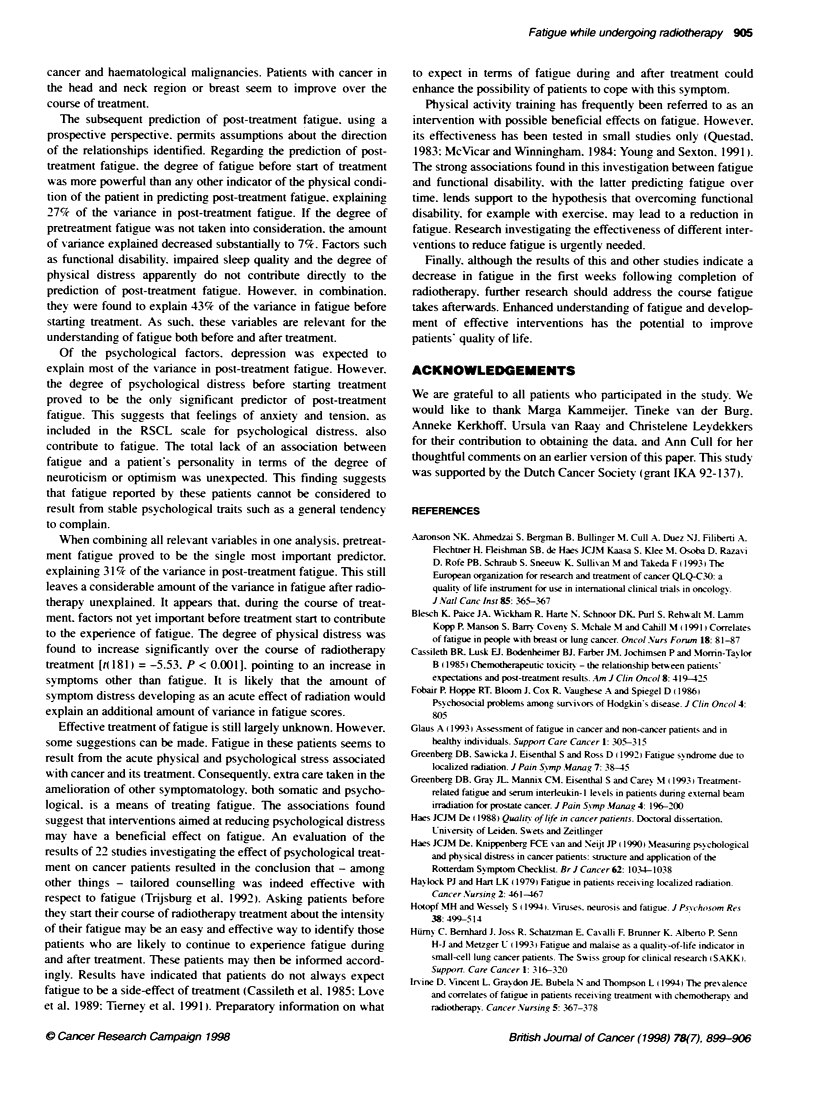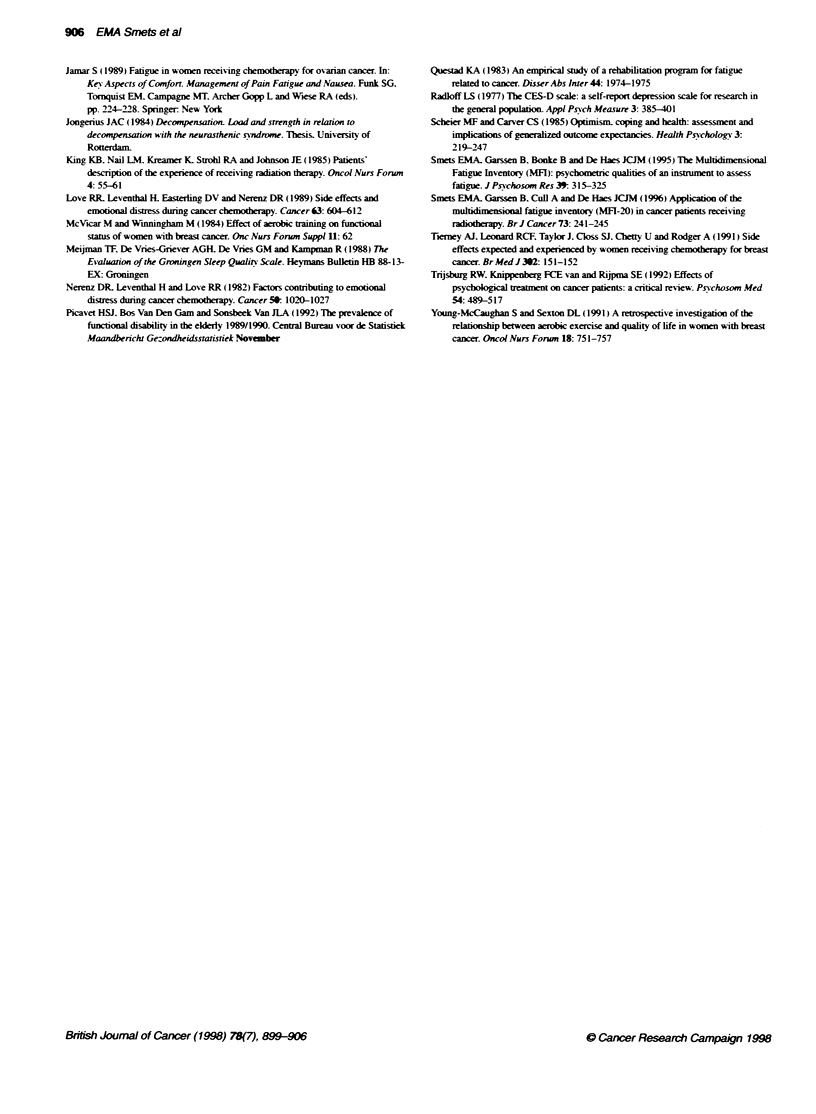# Fatigue and radiotherapy: (A) experience in patients undergoing treatment.

**DOI:** 10.1038/bjc.1998.599

**Published:** 1998-10

**Authors:** E. M. Smets, M. R. Visser, A. F. Willems-Groot, B. Garssen, F. Oldenburger, G. van Tienhoven, J. C. de Haes

**Affiliations:** Department of Medical Psychology, Academic Medical Centre, University of Amsterdam, The Netherlands.

## Abstract

Cancer patients undergoing radiotherapy frequently report fatigue. However, knowledge of the importance of fatigue for these patients and of the factors associated with their fatigue is limited. The aim of the current investigation was to gain more insight into fatigue as related to radiotherapy by answering the following questions. First, how is the experience of fatigue best described? Secondly, to what extent is fatigue related to sociodemographic, medical (including treatment), physical and psychological factors? Finally, is it possible to predict which patients will suffer from fatigue after completion of radiotherapy? Patients with different types of cancer receiving radiotherapy with curative intent (n = 250) were interviewed before and within 2 weeks of completion of radiotherapy. During treatment, patients rated their fatigue at 2-weekly intervals. Results indicate a gradual increase in fatigue over the period of radiotherapy and a decrease after completion of treatment. Fatigue scores obtained after radiotherapy were only slightly, although significantly, higher than pretreatment scores. After treatment, 46% of the patients reported fatigue among the three symptoms that caused them most distress. Significant associations were found between post-treatment fatigue and diagnosis, physical distress, functional disability, quality of sleep, psychological distress and depression. No association was found between fatigue and treatment or personality characteristics. Multivariate regression analysis demonstrated that the intensity of pretreatment fatigue was the best predictor of fatigue after treatment. In view of this finding, a regression analysis was performed to gain more insight into the variables predicting pretreatment fatigue. The degree of functional disability and impaired quality of sleep were found to explain 38% of the variance in fatigue before starting radiotherapy. Fatigue in disease-free patients 9 months after treatment is described in paper (B) in this issue.


British Journal of Cancer (1998) 78(7). 899–906
© 1998 Cancer Research Campaign

### Fatigue and radiotherapy: (A) experience in patients undergoing treatment

**EMA Smets[1], MRM Visser[1], AFMN Willems-Groot[1], B Garssen[2], F Oldenburger[3], G van Tienhoven[3] and JCJM de Haes[1]**

[1]Department of Medical Psychology, Academic Medical Centre, University of Amsterdam, PO Box 22700, 1100 DE Amsterdam, The Netherlands; [2]The Helen Dowling Institute for Biopsychosocial Medicine, Rotterdam; [3]Department of Radiotherapy, Academic Medical Centre, University of Amsterdam, Amsterdam, The Netherlands

**Summary** Cancer patients undergoing radiotherapy frequently report fatigue. However, knowledge of the importance of fatigue for these patients and of the factors associated with their fatigue is limited. The aim of the current investigation was to gain more insight into fatigue as related to radiotherapy by answering the following questions. First, how is the experience of fatigue best described? Secondly, to what extent is fatigue related to sociodemographic, medical (including treatment), physical and psychological factors? Finally, is it possible to predict which patients will suffer from fatigue after completion of radiotherapy? Patients with different types of cancer receiving radiotherapy with curative intent ($n = 250$) were interviewed before and within 2 weeks of completion of radiotherapy. During treatment, patients rated their fatigue at 2-weekly intervals. Results indicate a gradual increase in fatigue over the period of radiotherapy and a decrease after completion of treatment. Fatigue scores obtained after radiotherapy were only slightly, although significantly, higher than pretreatment scores. After treatment, 46% of the patients reported fatigue among the three symptoms that caused them most distress. Significant associations were found between post-treatment fatigue and diagnosis, physical distress, functional disability, quality of sleep, psychological distress and depression. No association was found between fatigue and treatment or personality characteristics. Multivariate regression analysis demonstrated that the intensity of pretreatment fatigue was the best predictor of fatigue after treatment. In view of this finding, a regression analysis was performed to gain more insight into the variables predicting pretreatment fatigue. The degree of functional disability and impaired quality of sleep were found to explain 38% of the variance in fatigue before starting radiotherapy. Fatigue in disease-free patients 9 months after treatment is described in paper (B) in this issue.

**Keywords:** fatigue; radiotherapy; psychological factor; physical factor; prediction

In oncology, there is growing awareness that, with the development of new treatment options the treatment burden for patients may be increased, with little or no improvement in survival. Consequently, the patients' appreciation of their quality of life following treatment is more frequently taken into account along with the more traditional outcomes of length of survival and morbidity.

Symptom distress is an important component of patients' overall evaluation of their well-being (e.g. de Haes, 1988). Fatigue is one of the common symptoms found to be negatively associated with patients' assessment of their quality of life (Aaronson et al, 1993; Hürny et al, 1993). Yet, despite its apparent importance, knowledge of the prevalence and correlates of fatigue is still limited.

In patients receiving radiotherapy, fatigue or tiredness is frequently reported. The experience of fatigue appears to be treatment related, as reflected by differences in prevalence rates between groups with different radiation fields, by a gradual increase in fatigue over the course of treatment and by a reduction in fatigue scores over weekends, when no treatment is given (King et al, 1985; Greenberg et al, 1992; Irvine et al, 1994). Fatigue during radiotherapy may result directly from radiation, but may also be an expression of the disease process or a residual effect of previous treatment.

Physical factors investigated to explain radiation-related fatigue include haematocrit and haemoglobin (Greenberg et al, 1992; Glaus, 1993; Irvine et al, 1994), weight and change in weight (Haylock and Hart, 1979; Greenberg et al, 1992; Glaus, 1993; Irvine et al, 1994), serum interleukin 1 (IL-1) (Greenberg et al, 1993), reverse triiodothyronine and pulse change with orthostatic stress (Greenberg et al, 1992). Except for change in weight (Haylock and Hart, 1979; Irvine et al, 1994), none of these factors was found to be significantly associated with fatigue. The distress associated with symptoms such as pain, nausea or sleep disturbances was found to be related to fatigue (Irvine et al, 1994).

So far, no studies have investigated the relation between psychological factors and fatigue in radiation patients. Studies investigating psychological distress in other cancer patients suggest a relation between fatigue and depression and anxiety (Nerenz et al, 1982; Fobair et al, 1986; Jamar, 1989; Blesh et al, 1991). This association might, in turn, be attributable to an association of these emotions and fatigue with personality characteristics, such as neuroticism or optimism. A person's disposition may be related to fatigue by influencing coping reactions. Optimists are more likely to engage in active attempts to cope with a problem. Persons with a neurotic disposition are more likely to dwell upon their negative experiences, employ avoiding strategies and disengage from active coping (Scheier and Carver, 1985). Disposition

*Received 23 May 1996*
*Revised 17 March 1998*
*Accepted 1 April 1998*

*Correspondence to:* EMA Smets may also contribute to a person's tendency to self-monitor for symptoms. Neurotic individuals are more sensitive to and likely to report any bodily sensations, including fatigue (Hotopf and Wessely, 1994).

The primary aim of this study was to come to a better understanding of fatigue in patients receiving radiotherapy. The following questions guided the study. First, how can the experience of fatigue as related to radiotherapy be described? Secondly, to what degree is fatigue related to sociodemographic, medical (including treatment), physical and psychological factors? Finally, is it possible to predict who will suffer most from fatigue after completion of radiotherapy?

### METHOD

### Sample and data collection procedure

Cancer patients attending for radiotherapy treatment at the Academic Medical Centre in Amsterdam were approached. Eligible patients were aged 18 years or older, receiving treatment on an outpatient basis for cure or control of cancer rather than for palliation, free of malignancy in the central nervous system, not receiving chemotherapy and native Dutch.

The radiation oncologist introduced the study at the first consultation with written information describing the purpose and procedure of the investigation. Patients were later contacted by telephone by the researchers to ask for consent. Of the 308 eligible patients, 250 (81%) agreed to participate. Patients who declined participation were requested to rate the fatigue they experienced during the previous week, as a check for bias in the study sample.

Participants were interviewed at their homes approximately 2 weeks before the start of treatment and 2 weeks after completion of treatment. During the period of treatment, patients rated their fatigue at 2-weekly intervals.

### Instruments

Diagnosis, Karnofsky score, weight at the start of treatment and treatment variables including dose, fractionation and radiation area were obtained from the patients' medical records. Levels of haemoglobin or haematocrit outside the normal range were recorded over the period of treatment. The patients' prognosis in terms of 5-year survival probability was classified by the Dutch Cancer Registration Office as either less than 20%, 20–40%, 40–60%, 60–80% or greater than 80%.

The following data were collected on interview: medical history, frequency of fatigue (never, hardly ever, sometimes, most of the time or always), the time of most intense fatigue during the day (no clear pattern, early morning, noon, afternoon, late afternoon, evening or depending upon time of radiation), physical sensations associated with fatigue (muscle weakness, sweating, uncomfortable feeling in the chest, sore muscles, blurred sight and shortness of breath: with response categories not at all, a bit, moderate and very much), less fatigue on days without radiation (yes, no, don't know) and hours of sleep. At the post-treatment interview, patients were asked to compare their present degree of fatigue with fatigue before the start of treatment (more fatigue, the same, less fatigue).

In both the pre- and post-treatment interview, the following instruments were used to assess fatigue in two ways. Firstly, The Multidimensional Fatigue Inventory (MFI-20) was used, which is a self-report instrument consisting of five scales based on different modes of expressing fatigue. 'General fatigue' includes general statements concerning a person's functioning such as 'I feel fit'. 'Physical fatigue' refers to the physical sensation related to the feeling of tiredness. Possible somatic symptoms of fatigue such as light-headedness or sore muscles are not included in this scale in order to exclude as much possible contamination with the symptoms of somatic illness, independent of fatigue. Reduction in activities and lack of motivation to start any activity are covered by the scales 'reduced activity' and 'reduced motivation' respectively. Each scale contains four items, with a five-point response format. Finally, cognitive symptoms such as having difficulties concentrating are included in the scale for 'mental fatigue' (Smets et al, 1995). Secondly, a single numerical rating scale ranging from 0 (not tired at all) to 10 (worst tiredness imaginable), was used, both in the interviews and for the 2-weekly assessment of fatigue.

Similar numerical rating scales were used to assess the patient's global assessment of his or her quality of life and the intensity of pain.

Functional disability was assessed by the Activities of Daily Living Questionnaire (Picavet et al, 1992), extended to cover habitual activities that may require effort but are not essential for self-care, including physical exercise, household activities, social activities, work related activities and mental activities.

Quality of sleep was measured using the general version of the Groningen Sleep Quality Scale (Meijman et al, 1988). Physical and psychological distress were assessed with the Rotterdam Symptom Checklist (RSCL; de Haes et al, 1990).

Depression was measured using The Centre for Epidemiologic Studies Depression Scale (CES-D; Radlof, 1977).

Finally, for the assessment of neuroticism and optimism the shortened version of the Dutch Personality Questionnaire (Jongerius, 1984) and the Life Orientation Test (LOT; Scheier and Carver, 1985) were used respectively. In contrast to the other instruments, these personality characteristics were only assessed before treatment.

### Statistical methods

The MFI scale of general fatigue was used as the dependent variable in all analyses involving associations with or prediction of fatigue. Hereafter, general fatigue is referred to as 'fatigue'. The scale for general fatigue was preferred over the use of the numerical rating scale because of its more favourable psychometric properties (Smets et al, 1995, 1996).

Paired *t*-tests were applied to test for changes in MFI fatigue scores over the period of radiotherapy treatment. The 2-weekly data from the numerical rating scale were subjected to MANOVA analyses for repeated measures.

To investigate bivariate associations, Pearson correlation coefficients or analyses of variance were used. For the prediction of post-treatment fatigue stepwise regression analyses were performed. The predictor variables were grouped to cover the following domains: sociodemographical, medical, treatment-related, physical and psychological. For each of these domains, a separate stepwise regression analysis was performed. Only predictors explaining a significant amount of the variance in fatigue were included in an overall regression analysis. Variables measured at nominal level, such as diagnosis, were entered as binary (yes/no) variables in the regression analyses.

© Cancer Research Campaign 1998

Plausible interactions, particularly involving variables for which no initial bivariate association with fatigue was found, were explored using scattergrams and partial correlations.

In order to avoid spurious associations between fatigue, depression and physical distress, because of similarity in item content, analyses were performed without overlapping items.

The associations between fatigue on the one hand and treatment dose and fractionation on the other were assessed for patients who received radiation at one target area only ($n$ = 198) because too few patients were radiated at two or more areas for statistical analyses. The relationships were determined separately for patients radiated on the head and neck ($n$ = 24), thorax ($n$ = 71) and abdomen/pelvis ($n$ = 118).

### RESULTS

### The sample

In Table 1, sociodemographic and medical information for the 250 participating patients is presented. Fourteen patients (6%) who were not available for pretreatment assessment agreed to complete the subsequent assessments. Of the 578 forms that were sent out for the 2-weekly assessment, 21 (4%) were not returned. After treatment, 216 of the original 250 patients (86%) were still on study; nine patients (4%) had declined further participation and 25 patients (10%) were not included in the second assessment for medical reasons such as receiving additional chemotherapy or because they could not be interviewed within the time limit of 1 month after treatment.

Patients who declined considered participation to be emotionally disturbing (10%), were too tired (5%) or too busy (9%), resented being interviewed at home (10%) or reported other reasons; 25% gave no reason.

Non-participants (19%) were found to be older (69.5 vs 64 year; $t$ = −2.98, d.f. = 288, $P$ < 0.005) and to have higher numerical fatigue scores (mean 4.7, s.d. 3.0) than participants (mean 3.6, s.d. 2.9; $t$ = −1.98, d.f. = 263, $P$ < 0.05). No differences were found with respect to gender distribution.

**Table 1** Sample characteristics ($n$ = 250)

| | | | | |
|---|---|---|---|---|
| | **Mean age** | **64 years ± 13** | | |
| | **Mean time since diagnosis** | **5.5 months ± 3** | | |
| | | ***n*** | **%** | **Range of total radiation dose (Gy)[a]** |
| Gender | Female | 103 | 42 | |
| | Male | 147 | 58 | |
| Education level | Less than high school | 53 | 23 | |
| | Lower educational level | 80 | 34 | |
| | High school | 62 | 26 | |
| | Advanced graduate degrees | 41 | 17 | |
| Marital status | Married | 185 | 74 | |
| | Living together | 13 | 5 | |
| | Single | 22 | 9 | |
| | Widowed | 29 | 12 | |
| Diagnosis | Head and neck | 15 | 6 | 60–66 |
| | Gastrointestinal | 13 | 6 | 45–60 |
| | Gynaecological | 31 | 12 | 40–70 |
| | Lung | 26 | 10 | 50–60 |
| | Breast | 47 | 19 | 50–75 |
| | Prostate | 64 | 26 | 60–70 |
| | Testis | 7 | 3 | 26 |
| | Other genitourinary tract | 22 | 9 | 40–70 |
| | Haematological malignancies | 18 | 7 | 40 |
| | Miscellaneous | 6 | 2 | 40–70 |
| Karnofsky score | 50 | 2 | 1 | |
| | 60 | 2 | 1 | |
| | 70 | 5 | 2 | |
| | 80 | 33 | 13 | |
| | 90 | 84 | 34 | |
| | 100 | 106 | 42 | |
| Five-year survival probability | <20% | 27 | 11 | |
| | 20–40% | 17 | 7 | |
| | 40–60% | 29 | 12 | |
| | 60–80% | 76 | 30 | |
| | >80% | 53 | 21 | |
| Co-morbidity | | 123 | 52 | |

[a]Variation in dose schemes within the tumour groups is due to variations in indications; e.g. post-operative adjuvant vs primary radiotherapeutic treatment.

### Course and description of fatigue

#### *Fatigue over the course of treatment*

Table 2 contains the average pre- and post-treatment scores on the MFI subscales. General fatigue scores increased significantly over treatment ($t$ = −2.54, d.f. = 199, $P$ < 0.05), whereas mental fatigue tended to decrease ($t$ = 1.90, d.f. = 200, $P$ = 0.059). No significant differences were found for the other three scales.

In Figure 1 the course of fatigue over the time of treatment is shown for patients with a treatment period of 2–4 weeks, 4–6 weeks or 6–8 weeks respectively. These data from the numerical rating scale demonstrate an increase in fatigue over the course of treatment and a decline after finishing treatment, independent of the duration of treatment. MANOVAs indicated these changes to be significant for all three groups (group 1, $F(3, 25)$ = 39, 19, $P$ < 0.001; group 2, $F(4,55)$ = 56.70, $P$ < 0.001; group 3, $F(5,97)$ = 54.87, $P$ < 0.001)

#### *Post-treatment description of the fatigue experience*

During the period of treatment, 40% of all patients reported being tired most of the time, 33% were sometimes and 27% hardly ever tired. When comparing their post-treatment level of fatigue with fatigue before treatment, 44% of the patients reported an increase, 26% a decrease and 30% no change. Percentages in the remainder of this section are based on the 166 patients who had post-treatment fatigue scores greater than 1 on the numerical rating scale. The time of most intense fatigue was shortly after their daily radiation treatment for 20% of patients, whereas 37% reported no clear pattern. For all other responses related to timing, percentages were less than 15% each. Twenty-eight per cent of patients reported being less tired on days without radiation. Concerning associated physical symptoms, shortness of breath and sweating were both reported by 29% of the patients (the response options 'a bit', 'moderate' and 'very much' combined), muscle weakness by 20%, muscle soreness by 19%, uncomfortable feeling at the chest by 16% and blurred vision by 13%.

After treatment, 29% of patients rated their fatigue on the RSCL as 'moderate' and 17% as 'very much'. For 46%, fatigue was reported as one of the three symptoms that caused them most

© Cancer Research Campaign 1998

**Table 2** Mean scores on the separate MFI scales at pre- and post-treatment (range for each scale: 4–20)

| | Pretreatment ($n$ = 230) | | Post-treatment ($n$ = 216) | |
|---|---|---|---|---|
| | Mean | s.d. | Mean | s.d. |
| General fatigue | 11.00 | 5.70 | 11.68[a] | 5.86 |
| Physical fatigue | 11.15 | 4.92 | 11.71 | 5.25 |
| Reduced activity | 11.93 | 5.11 | 11.69 | 5.25 |
| Reduced motivation | 8.83 | 4.77 | 8.73 | 4.80 |
| Mental fatigue | 8.30 | 4.87 | 7.55 | 4.82 |

[a]Significant difference compared with pretreatment, $P < 0.05$.

**Figure 1** Course of fatigue over the period of radiotherapy treatment. Numerical fatigue scores (range 0–10). T1, pretreatment assessment; T2, post-treatment assessment; group 1, 31 patients receiving 2–4 weeks of radiotherapy; group 2, 74 patients receiving 4–6 weeks of radiotherapy; group 3, 120 patients receiving 6–8 weeks of radiotherapy. The bars indicate mean scores, the lines 1 standard deviation. ■, Group 1; ⬚, group 2; ▨, group 3

distress. No other symptom from the RSCL was reported with such a high prevalence. Fatigue correlated –0.46 ($P < 0.001$) with the patient's overall quality of life, thus explaining 21% of the variance in overall quality of life.

### Bi-variate associations with post-treatment fatigue

None of the sociodemographic variables was found to be related to post-treatment fatigue (see Table 3). Of the medical variables, only diagnosis was associated with fatigue. Lung cancer patients reported most (mean 15.0, s.d. 5.7) and patients with malignancies in the head and neck region least (mean 10.5, s.d. 6.3) fatigue after treatment (see Figure 2). Paired $t$-tests were performed for the four largest diagnostic groups (gynaecological cancer, lung cancer, breast cancer and urogenital malignancies) and a significant increase was found for the group of patients with urogenital malignancies only ($t$ = –3.09, d.f. = 77, $P < 0.005$).

No relationships were found between post-treatment fatigue and the radiotherapy characteristics of radiation dose or fractionation. Also, no difference emerged in post-treatment fatigue between women with breast cancer who did ($n$ = 15; mean 11.0, s.d. 6.2) or did not ($n$ = 24; mean 11.8, s.d. 5.7) receive treatment with brachytherapy.

Of the indicators of physical functioning, weight before starting treatment and measures for anaemia were unrelated to fatigue scores. The total degree of symptom distress proved to be associated with fatigue, as did pain intensity, sleep disturbances, number of hours of sleep at night and day-time napping. Finally, the more patients were impaired in their capacity to perform daily activities, the higher their fatigue scores.

Fatigue and psychological state were related as follows: psychological distress in general and depression in particular were related to post-treatment fatigue, whereas neuroticism and optimism were not.

### Prediction of post-treatment fatigue

Results regarding the prediction of post-treatment fatigue from patient characteristics at pretreatment and radiotherapy aspects are presented in Table 4. None of the sociodemographic characteristics of patients (age, gender, education) predicted post-treatment fatigue. Of the medical variables (diagnosis, prognosis and co-morbidity), a diagnosis of lung cancer explained 3% of the variance in post-treatment fatigue scores. Of the radiation treatment variables (total radiation dose and number of fractions), the total radiation dose explained 2% of the variance in post-treatment fatigue.

For the domain of physical predictors assessed pretreatment (weight, functional disability, sleep, physical distress, pain and fatigue), the following interactions were considered for inclusion in the regression analysis. It was assumed that, although prognosis and co-morbidity per se appeared to be unrelated to fatigue, they might interact with the degree of physical distress. Physical distress in combination with an unfavourable prognosis, or with co-morbidity, might explain an additional amount of the variance in post-treatment fatigue. Indeed, the data suggested that the association between the degree of physical distress and post-treatment fatigue was different for the separate prognostic groups. There were no indications of a similar interaction with co-morbidity. Therefore, only the former interaction term was included in the analysis. In addition, it was hypothesized that physical distress and functional disability would be differently associated with fatigue, depending on the patient's age. However, there was no evidence supporting the latter hypothesis and therefore the interaction with age was not included in the regression analysis. From the analysis, fatigue before the start of radiotherapy proved to be the best predictor, explaining 27% of the variance in post-treatment fatigue. None of the other variables in this domain, including the interaction term, improved the prediction. The same analysis, with pretreatment fatigue excluded, resulted in 7% of the variance being explained by the patients' degree of functional disability.

© Cancer Research Campaign 1998

**Table 3** Bivariate associations of the various variables with post-treatment general fatigue scores using Pearson product moment correlations ($r$) or analyses of variance ($F$)

| | Post-treatment fatigue | |
|---|---|---|
| **Domains and their variables** | **Statistics** | ***P*** |
| *Sociodemographical* | | |
| Sex[a] | $F(1, 214) = 1.80$ | NS |
| Age[a] | $r = -0.08$ | NS |
| Education[a] | $F(3, 144) = 0.10$ | NS |
| *Medical* | | |
| Diagnosis[a] | $F(6, 203) = 2.16$ | <0.05 |
| Prognosis[a] | $r = -0.13$ | NS |
| Co-morbidity[a] | $F(1, 199) = 0.23$ | NS |
| *Radiotherapy* | | |
| Dose[b] | $r = -0.23, -0.25, -0.04$[d] | NS |
| Fractionations[b] | $r = -0.05, -0.29, -0.03$[d] | NS |
| Brachytherapy[b] | $F(1,37) = 0.16$ | NS |
| *Physical* | | |
| Physical distress[b] | $r = 0.53$ | <0.001 |
| Pain[b] | $r = 0.36$ | <0.001 |
| Quality of sleep[b] | $r = 0.41$ | <0.001 |
| Hours of sleep[b] | $r = 0.26$ | <0.001 |
| Day-time napping[b] | $F(1, 214) = 22.82$ | <0.001 |
| Weight[a] | $r = 0.01$ | NS |
| Haemoglobin[c] | $F(1, 191) = 0.39$ | NS |
| Haematocrit[c] | $F(1, 190) = 0.01$ | NS |
| Functional disability[c] | $r = 0.60$ | <0.001 |
| *Psychological* | | |
| Psychological distress[b] | $r = 0.37$ | <0.001 |
| Depression[b] | $r = 0.43$ | <0.001 |
| Optimism[a] | $r = -0.08$ | NS |
| Neuroticism[a] | $r = 0.08$ | NS |

NS, not significant. [a]Assessed before radiotherapy. [b]Assessed after radiotherapy. [c]Assessed during radiotherapy. [d]For patients radiated on the head and neck ($n = 24$), thorax ($n = 71$) and abdomen/pelvis ($n = 118$) respectively. In each of the analyses the degrees of freedom vary slightly because of listwise deletion.

Regarding the psychological variables (neuroticism, optimism, psychological distress and depression) it was assumed that neuroticism and optimism might be associated with fatigue in a different way for men and women. In addition, it was hypothesized that disposition might interact with the degree of physical distress, functional disability or reported quality of sleep. However, prior exploration yielded no evidence to justify the inclusion of these interaction terms in the analysis. The analysis for this domain showed psychological distress to explain 5% of the variance in fatigue after treatment.

As a result of the foregoing analyses by domain, a subsequent overall analysis included a diagnosis of lung cancer, the degree of pretreatment fatigue and psychological distress and the total radiation dose. Thirty-one per cent of the variance in post-treatment fatigue was found to be explained by pretreatment fatigue only. A second analysis was performed, with pretreatment fatigue excluded. Of the variance after treatment, 17% was explained by the patients' functional disability at pretreatment assessment and the diagnosis of lung cancer.

### Prediction of pretreatment fatigue

As a result of the foregoing analyses, it became apparent that pretreatment fatigue is the single best predictor of the degree of fatigue after treatment. In view of this, it became of interest to investigate which factors contribute to pretreatment fatigue.

**Figure 2** Mean pre- and post-treatment general fatigue scores for the various diagnostic subgroups (range 4–20) The bars indicate mean scores, the lines 1 standard deviation. ■, Pretreatment; ▧, post-treatment

Therefore, similar regression analyses were performed, now using pretreatment fatigue as dependent variable. Results are presented in Table 5.

The domain-specific analyses indicated the following factors as being related to higher pretreatment fatigue: being female, not having a diagnosis of urogenital cancer, a higher degree of functional disability and physical distress, impaired quality of sleep and a higher degree of depression. When combined in one analysis the degree of functional disability and impaired quality of sleep remained significant predictors, explaining 38% of the variance in fatigue.

### DISCUSSION

Two weeks after the end of radiotherapy treatment 40% of the patients reported having been almost continuously tired during the treatment period, 44% reported that fatigue had increased over this period and fatigue was among the three most distressing symptoms for almost half of these patients. In addition, fatigue was found to explain 21% of patients' overall rating of their post-treatment quality of life. This is a considerable amount of variance explained by a single symptom. Together, these findings illustrate the importance of fatigue for patients. As a consequence, they indicate that fatigue deserves attention in radiotherapy treatment.

The prevalence and impact of fatigue as found in this investigation may underestimate the actual problem. The differences in age and fatigue between participants and non-participants suggest a selection bias, with the older and more tired patients being more inclined to refuse participation. Another potential source of error is bias as a result of loss to follow-up. Although attrition between the two assessment points was small (14%), it involved mostly patients with complications of their disease or treatment.

The gradual increase in fatigue over the course of treatment, followed by a decrease after ending treatment as demonstrated in Figure 1, suggests an acute effect of radiotherapy on fatigue. This finding is in line with results reported by others (King et al, 1985; Greenberg et al, 1992; Irvine et al, 1994). Other indicators of an acute radiation effect are the weekend effect reported by 28% of the patients, and the finding that 20% reported fatigue to have been most intense shortly after being radiated.

© Cancer Research Campaign 1998

**Table 4** Significant pretreatment predictors of post-treatment general fatigue scores, using stepwise regression analyses

| Domain | Predictor | *R* | $R^2$ | Regression coefficient | | |
|---|---|---|---|---|---|---|
| | | | | B | s.e.B | *P* |
| 1. Medical | Lung cancer | 0.18 | 0.03 | 3.57 | 1.36 | <0.01 |
| 2. Radiation treatment | Total dose | 0.15 | 0.02 | −0.04 | 0.02 | <0.05 |
| 3. Physical | Pretreatment fatigue | 0.52 | 0.27 | 0.53 | 0.08 | <0.0001 |
| 3a. Physical without pre-treatment fatigue | Functional disability | 0.27 | 0.07 | 0.17 | 0.06 | <0.005 |
| 4. Psychological | Psychological distress | 0.23 | 0.05 | 0.35 | 0.11 | <0.01 |
| Combined (1,2,3,4) | Pretreatment fatigue | 0.56 | 0.31 | 0.57 | 0.06 | <0.0001 |
| Combined without pretreatment fatigue | Functional disability | 0.38 | 0.15 | 0.22 | 0.04 | <0.0001 |
| (1,2,3a,4) | Lung cancer | 0.03 | 0.02 | 3.34 | 1.66 | <0.05 |

**Table 5** Significant pretreatment predictors of pretreatment general fatigue scores, using stepwise regression analyses

| Domain | Predictor | *R* | $R^2$ | Regression coefficient | | |
|---|---|---|---|---|---|---|
| | | | | B | s.e.B | *P* |
| Sociodemographic | Gender | 0.21 | 0.04 | 2.37 | 0.75 | <0.005 |
| Medical | Urogenital cancer | 0.25 | 0.06 | −2.85 | 0.80 | <0.0005 |
| Physical | Functional disability | 0.57 | 0.33 | 0.24 | 0.05 | <0.0001 |
| | Sleep quality | 0.06 | 0.07 | 0.43 | 0.13 | <0.001 |
| | Physical distress | 0.02 | 0.03 | 0.20 | 0.10 | <0.05 |
| Psychological | Depression | 0.35 | 0.12 | 0.36 | 0.07 | <0.0001 |
| Combined | Functional disability | 0.56 | 0.31 | 0.28 | 0.04 | <0.0001 |
| | Sleep quality | 0.06 | 0.07 | 0.50 | 0.11 | <0.0001 |

In view of these indications of a radiation effect, it is somewhat surprising that treatment characteristics such as radiation dose and fractionation were almost unrelated to fatigue. A similar result has been reported by Irvine et al (1994). It should be noted, however, that both studies involved very heterogeneous samples. In this investigation, crude categorizations were used (e.g. radiation target area: head and neck region, thorax and abdomen/pelvis) to have large enough groups for meaningful statistical analyses. Studies involving more homogeneous samples with respect to diagnosis and/or treatment, such as in clinical trials, might provide more insightful information on the role of specific radiotherapy characteristics in fatigue.

Although an increase in general fatigue scores is found over the treatment period, the numerical fatigue scores (Figure 1) showed a lack of difference between pre- and post-treatment. This discrepancy indicates that the MFI scale for general fatigue is more sensitive in detecting change over time than the single numerical scale. It also suggests that, although significant, the difference in fatigue between the two moments of assessment is small. At two weeks after completion of radiotherapy, fatigue has already decreased to a level only slightly higher than before the start of treatment.

The lack of more substantial differences in fatigue before and after radiotherapy does not, however, exclude a radiation treatment effect. One would have expected fatigue to decline after initial treatment, mostly surgery, if not followed by radiotherapy. Instead, fatigue increased for the group as a whole, suggesting that radiotherapy at least postpones the process of recovery for some patients.

It also deserves mentioning that both pre- and post-treatment scores were significantly higher than MFI fatigue scores from a random sample from the Dutch general population ($n$ = 139): [mean fatigue score = 9.91 (s.d. 5.2); difference with patients' pretreatment fatigue: $F(360,1) = 5.24$, $P < 0.05$; difference with post-treatment fatigue: $F(346,1) = 15.52$, $P < 0.001$); (for more detailed information, see following paper].

An important question addressed in this study involved the factors associated with fatigue. Bivariate associations were assessed first, yielding multiple associations with both medical, physical and psychological variables. The direction of these associations is not always straightforward. For example, an impaired quality of sleep is most likely to lead to more fatigue, which causes a person to spend more time in bed. This, in turn, might aggravate the sleeping problems. Other associations may indicate that fatigue increases the burden induced by other symptoms, contributes to impaired performance of daily activities and causes a person to feel anxious or depressed. However, converse relations are also possible.

The association with diagnosis indicates that patients from different diagnostic subgroups differ significantly in the degree of post-treatment fatigue. Cancer of the lungs causes a person to feel more fatigued than cancer in the head and neck region. The results with respect to the diagnostic subgroups, as presented in Figure 2, should be interpreted with reservation, because of the small numbers involved. They seem to indicate that the increase in fatigue scores over treatment might be ascribed primarily to patients with gynaecological cancer, lung cancer, uro-genital

© Cancer Research Campaign 1998

cancer and haematological malignancies. Patients with cancer in the head and neck region or breast seem to improve over the course of treatment.

The subsequent prediction of post-treatment fatigue, using a prospective perspective, permits assumptions about the direction of the relationships identified. Regarding the prediction of post-treatment fatigue, the degree of fatigue before start of treatment was more powerful than any other indicator of the physical condition of the patient in predicting post-treatment fatigue, explaining 27% of the variance in post-treatment fatigue. If the degree of pretreatment fatigue was not taken into consideration, the amount of variance explained decreased substantially to 7%. Factors such as functional disability, impaired sleep quality and the degree of physical distress apparently do not contribute directly to the prediction of post-treatment fatigue. However, in combination, they were found to explain 43% of the variance in fatigue before starting treatment. As such, these variables are relevant for the understanding of fatigue both before and after treatment.

Of the psychological factors, depression was expected to explain most of the variance in post-treatment fatigue. However, the degree of psychological distress before starting treatment proved to be the only significant predictor of post-treatment fatigue. This suggests that feelings of anxiety and tension, as included in the RSCL scale for psychological distress, also contribute to fatigue. The total lack of an association between fatigue and a patient's personality in terms of the degree of neuroticism or optimism was unexpected. This finding suggests that fatigue reported by these patients cannot be considered to result from stable psychological traits such as a general tendency to complain.

When combining all relevant variables in one analysis, pretreatment fatigue proved to be the single most important predictor, explaining 31% of the variance in post-treatment fatigue. This still leaves a considerable amount of the variance in fatigue after radiotherapy unexplained. It appears that, during the course of treatment, factors not yet important before treatment start to contribute to the experience of fatigue. The degree of physical distress was found to increase significantly over the course of radiotherapy treatment [$t(181) = -5.53$, $P < 0.001$], pointing to an increase in symptoms other than fatigue. It is likely that the amount of symptom distress developing as an acute effect of radiation would explain an additional amount of variance in fatigue scores.

Effective treatment of fatigue is still largely unknown. However, some suggestions can be made. Fatigue in these patients seems to result from the acute physical and psychological stress associated with cancer and its treatment. Consequently, extra care taken in the amelioration of other symptomatology, both somatic and psychological, is a means of treating fatigue. The associations found suggest that interventions aimed at reducing psychological distress may have a beneficial effect on fatigue. An evaluation of the results of 22 studies investigating the effect of psychological treatment on cancer patients resulted in the conclusion that – among other things – tailored counselling was indeed effective with respect to fatigue (Trijsburg et al, 1992). Asking patients before they start their course of radiotherapy treatment about the intensity of their fatigue may be an easy and effective way to identify those patients who are likely to continue to experience fatigue during and after treatment. These patients may then be informed accordingly. Results have indicated that patients do not always expect fatigue to be a side-effect of treatment (Cassileth et al, 1985; Love et al, 1989; Tierney et al, 1991). Preparatory information on what to expect in terms of fatigue during and after treatment could enhance the possibility of patients to cope with this symptom.

Physical activity training has frequently been referred to as an intervention with possible beneficial effects on fatigue. However, its effectiveness has been tested in small studies only (Questad, 1983; McVicar and Winningham, 1984; Young and Sexton, 1991). The strong associations found in this investigation between fatigue and functional disability, with the latter predicting fatigue over time, lends support to the hypothesis that overcoming functional disability, for example with exercise, may lead to a reduction in fatigue. Research investigating the effectiveness of different interventions to reduce fatigue is urgently needed.

Finally, although the results of this and other studies indicate a decrease in fatigue in the first weeks following completion of radiotherapy, further research should address the course fatigue takes afterwards. Enhanced understanding of fatigue and development of effective interventions has the potential to improve patients' quality of life.

### ACKNOWLEDGEMENTS

We are grateful to all patients who participated in the study. We would like to thank Marga Kammeijer, Tineke van der Burg, Anneke Kerkhoff, Ursula van Raay and Christelene Leydekkers for their contribution to obtaining the data, and Ann Cull for her thoughtful comments on an earlier version of this paper. This study was supported by the Dutch Cancer Society (grant IKA 92-137).

### REFERENCES

Aaronson NK, Ahmedzai S, Bergman B, Bullinger M, Cull A, Duez NJ, Filiberti A, Flechtner H, Fleishman SB, de Haes JCJM Kaasa S, Klee M, Osoba D, Razavi D, Rofe PB, Schraub S, Sneeuw K, Sullivan M and Takeda F (1993) The European organization for research and treatment of cancer QLQ-C30: a quality of life instrument for use in international clinical trials in oncology. *J Natl Canc Inst* **85**: 365–367

Blesch K, Paice JA, Wickham R, Harte N, Schnoor DK, Purl S, Rehwalt M, Lamm Kopp P, Manson S, Barry Coveny S, Mchale M and Cahill M (1991) Correlates of fatigue in people with breast or lung cancer. *Oncol Nurs Forum* **18**: 81–87

Cassileth BR, Lusk EJ, Bodenheimer BJ, Farber JM, Jochimsen P and Morrin-Taylor B (1985) Chemotherapeutic toxicity – the relationship between patients' expectations and post-treatment results. *Am J Clin Oncol* **8**: 419–425

Fobair P, Hoppe RT, Bloom J, Cox R, Vaughese A and Spiegel D (1986) Psychosocial problems among survivors of Hodgkin's disease. *J Clin Oncol* **4**: 805

Glaus A (1993) Assessment of fatigue in cancer and non-cancer patients and in healthy individuals. *Support Care Cancer* **1**: 305–315

Greenberg DB, Sawicka J, Eisenthal S and Ross D (1992) Fatigue syndrome due to localized radiation. *J Pain Symp Manag* **7**: 38–45

Greenberg DB, Gray JL, Mannix CM, Eisenthal S and Carey M (1993) Treatment-related fatigue and serum interleukin-1 levels in patients during external beam irradiation for prostate cancer. *J Pain Symp Manag* **4**: 196–200

Haes JCJM De (1988) *Quality of life in cancer patients*. Doctoral dissertation. University of Leiden. Swets and Zeitlinger

Haes JCJM De, Knippenberg FCE van and Neijt JP (1990) Measuring psychological and physical distress in cancer patients: structure and application of the Rotterdam Symptom Checklist. *Br J Cancer* **62**: 1034–1038

Haylock PJ and Hart LK (1979) Fatigue in patients receiving localized radiation. *Cancer Nursing* **2**: 461–467

Hotopf MH and Wessely S (1994). Viruses, neurosis and fatigue. *J Psychosom Res* **38**: 499–514

Hürny C, Bernhard J, Joss R, Schatzman E, Cavalli F, Brunner K, Alberto P, Senn H-J and Metzger U (1993) Fatigue and malaise as a quality-of-life indicator in small-cell lung cancer patients. The Swiss group for clinical research (SAKK). *Support. Care Cancer* **1**: 316–320

Irvine D, Vincent L, Graydon JE, Bubela N and Thompson L (1994) The prevalence and correlates of fatigue in patients receiving treatment with chemotherapy and radiotherapy. *Cancer Nursing* **5**: 367–378

© Cancer Research Campaign 1998

Jamar S (1989) Fatigue in women receiving chemotherapy for ovarian cancer. In: *Key Aspects of Comfort. Management of Pain Fatigue and Nausea*. Funk SG, Tornquist EM, Campagne MT, Archer Gopp L and Wiese RA (eds). pp. 224–228. Springer: New York

Jongerius JAC (1984) *Decompensation. Load and strength in relation to decompensation with the neurasthenic syndrome*. Thesis. University of Rotterdam.

King KB, Nail LM, Kreamer K, Strohl RA and Johnson JE (1985) Patients' description of the experience of receiving radiation therapy. *Oncol Nurs Forum* 4: 55–61

Love RR, Leventhal H, Easterling DV and Nerenz DR (1989) Side effects and emotional distress during cancer chemotherapy. *Cancer* **63**: 604–612

McVicar M and Winningham M (1984) Effect of aerobic training on functional status of women with breast cancer. *Onc Nurs Forum Suppl* **11**: 62

Meijman TF, De Vries-Griever AGH, De Vries GM and Kampman R (1988) *The Evaluation of the Groningen Sleep Quality Scale*. Heymans Bulletin HB 88-13-EX: Groningen

Nerenz DR, Leventhal H and Love RR (1982) Factors contributing to emotional distress during cancer chemotherapy. *Cancer* **50**: 1020–1027

Picavet HSJ, Bos Van Den Gam and Sonsbeek Van JLA (1992) The prevalence of functional disability in the elderly 1989/1990. Central Bureau voor de Statistiek *Maandbericht Gezondheidsstatistiek* **November**

Questad KA (1983) An empirical study of a rehabilitation program for fatigue related to cancer. *Disser Abs Inter* **44**: 1974–1975

Radloff LS (1977) The CES-D scale: a self-report depression scale for research in the general population. *Appl Psych Measure* **3**: 385–401

Scheier MF and Carver CS (1985) Optimism, coping and health: assessment and implications of generalized outcome expectancies. *Health Psychology* **3**: 219–247

Smets EMA, Garssen B, Bonke B and De Haes JCJM (1995) The Multidimensional Fatigue Inventory (MFI): psychometric qualities of an instrument to assess fatigue. *J Psychosom Res* **39**: 315–325

Smets EMA, Garssen B, Cull A and De Haes JCJM (1996) Application of the multidimensional fatigue inventory (MFI-20) in cancer patients receiving radiotherapy. *Br J Cancer* **73**: 241–245

Tierney AJ, Leonard RCF, Taylor J, Closs SJ, Chetty U and Rodger A (1991) Side effects expected and experienced by women receiving chemotherapy for breast cancer. *Br Med J* **302**: 151–152

Trijsburg RW, Knippenberg FCE van and Rijpma SE (1992) Effects of psychological treatment on cancer patients: a critical review. *Psychosom Med* **54**: 489–517

Young-McCaughan S and Sexton DL (1991) A retrospective investigation of the relationship between aerobic exercise and quality of life in women with breast cancer. *Oncol Nurs Forum* **18**: 751–757

*© Cancer Research Campaign 1998*